# Community and Proteomic Analysis of Anaerobic Consortia Converting Tetramethylammonium to Methane

**DOI:** 10.1155/2017/2170535

**Published:** 2017-12-17

**Authors:** Wei-Yu Chen, Lucia Kraková, Jer-Horng Wu, Domenico Pangallo, Lenka Jeszeová, Bing Liu, Hidenari Yasui

**Affiliations:** ^1^Department of Environmental Engineering, National Cheng Kung University, No. 1, University Road, East District, Tainan City 701, Taiwan; ^2^Institute of Molecular Biology, Slovak Academy of Sciences, Dubravska Cesta 21, 84551 Bratislava, Slovakia; ^3^Faculty of Environmental Engineering, The University of Kitakyushu, 1-1 Hibikino, Wakamatsu, Kitakyushu 808-0135, Japan

## Abstract

Tetramethylammonium-degrading methanogenic consortia from a complete-mixing suspended sludge (CMSS) and an upflow anaerobic sludge blanket (UASB) reactors were studied using multiple PCR-based molecular techniques and shotgun proteomic approach. The prokaryotic 16S rRNA genes of the consortia were analyzed by quantitative PCR, high-throughput sequencing, and DGGE-cloning methods. The results showed that methanogenic *archaea* were highly predominant in both reactors but differed markedly according to community structure. Community and proteomic analysis revealed that *Methanomethylovorans* and *Methanosarcina* were the major players for the demethylation of methylated substrates and methane formation through the reduction pathway of methyl-S-CoM and possibly, acetyl-CoA synthase/decarbonylase-related pathways. Unlike high dominance of one *Methanomethylovorans* population in the CMSS reactor, diverse methylotrophic *Methanosarcina* species inhabited in syntrophy-like association with hydrogenotrophic *Methanobacterium* in the granular sludge of UASB reactor. The overall findings indicated the reactor-dependent community structures of quaternary amines degradation and provided microbial insight for the improved understanding of engineering application.

## 1. Introduction

Tetramethylammonium hydroxide ([(CH_3_)_4_N][OH]) is a developing fluid used in photolithography processes in semiconductor, thin-film transistor liquid crystal display, and light-emitting diode manufacturing industries. The waste stream generated usually contains high concentrations of tetramethylammonium (QMA) and has been treated by the anaerobic process in serially combined with the activated sludge process [1] or with the processes for the nitrogen removal [2, 3] to meet the discharge standards of carbon and nitrogen. Other than the advantages such as recovery of methane energy, low-nutrient requirements and less sludge production, the anaerobic process is a key to release ammonium, which efficacy greatly influences the performance of downstream aerobic/anoxic reactors. Because of its ability to manage high concentrations of methylated compounds and ammonia, the anaerobic process as a prior treatment unit can accommodate high organic loadings to minimize the land use for the treatment plant [4].

Under the obligately anaerobic conditions, QMA with four methyl moieties serves as an excellent substrate for methane production through methanogenesis pathways. In the growth of methylotrophic methanogens with QMA, it has been proposed that the electrons required for reduction of the methyl groups to methane are obtained from the oxidation of an additional methyl group to CO_2_ (1). During direct methylotrophic methanogenesis, the methyl groups of the compounds are transferred to the thiol group of coenzyme M (HS-CoM) by substrate-specific methyltransferases and then produce methane [5, 6]. Several marine methylotrophic methanogens under genera *Methanococcoides* and *Methanolobus* have been obtained in pure cultures to prove the direct methanogenesis from QMA [6–8]. In contrast to this mode of growth, the quaternary amines can be converted to methane in the cocultures of methanogens and sulfate-reducing bacteria [9]. Because the reaction from QMA degradation to H_2_ is energetically unfavorable (*Δ*G°′ of (2) is +319.9 kJ/reaction at the standard conditions of 1 M, 25°C, 1 atm, and pH 7), it requires the hydrogen-scavenging methanogenesis (3) or homoacetogenesis (4) as a coupling reaction to proceed. In the anaerobic reactor where hydrogen partial pressure is usually sufficiently low for syntrophic growth ((2) + (3)/(4)), the corresponding bacterial and archaeal populations can interact closely in a complex manner in the sludge consortia [10, 11]. Rather, complex microbial populations are expected to participate in the indirect methanogenesis route. However, the microbial community structure to the indirect route of QMA degradation has not been reported, and to date, relevant knowledge remains relatively limited.

(1) $$\begin{array}{c} \begin{array}{l} {\left({CH}_{3}\right)}_{4}N^{+}+3H_{2}O\to \\ 3CH_{4}+{{HCO}_{3}}^{-}+{{NH}_{4}}^{+}+H^{+}\;\left(\Delta G^{o\prime }=-86.2\;kJ/reaction\right) \end{array} \end{array}$$

(2) $$\begin{array}{c} \begin{array}{l} {\left({CH}_{3}\right)}_{4}N^{+}+12H_{2}O\to \\ 4HC{O_{3}}^{-}+12H_{2}+{{NH}_{4}}^{+}+4H^{+}\;\left(\Delta G^{o\prime }=+319.9\;kJ/reaction\right) \end{array} \end{array}$$

(3) $$\begin{array}{c} {{HCO}_{3}}^{-}+4H_{2}+H^{+}\to {CH}_{4}+3H_{2}O\;\left(\Delta G^{o\prime }=-135.6\;kJ/reaction\right) \end{array}$$

(4) $$\begin{array}{c} \begin{array}{l} 2HC{O_{3}}^{-}+4H_{2}+H^{+}\to \\ {CH}_{3}{COO}^{-}+4H_{2}O\;\left(\Delta G^{o\prime }=-104.6\;kJ/reaction\right) \end{array} \end{array}$$

Free energy change (*Δ*G°′) was calculated at the standard conditions (1 M, 1 atm, 25°C, and pH 7).

Various types of anaerobic reactors, such as upflow anaerobic blanket (UASB) [4] and complete-mixing suspended sludge (CMSS) [12] reactors, have been used for the treatment of QMA-containing wastewater. The two types of reactors differ in mixing conditions (complete mixing versus plug flow) and growth models (dispersed growth versus attached growth). Because reactor performance is highly associated with microbial activity, clarifying the relevant population structure and their nexus inside anaerobic reactors can provide key information for increasing the efficiency of organic matter decomposition and methane recovery, as well as improving reactor performance. In the present study, the sludge samples from QMA-degrading CMSS and UASB reactors are analyzed using multiple PCR-based techniques and the shotgun proteomic approach [13], with attempt to gain insight into QMA-degrading communities in the reactor environment and the metabolic pathways used by predominant populations.

## 2. Materials and Methods

### 2.1. Sludge Samples

Anaerobic sludge from a laboratory-scale CMSS system (sample CMJP) and a full-scale UASB reactor (samples UASB1a-UASB5a) was studied. The laboratory-scale CMSS system consisting of a 10 L gas-tight continuously stirred tank reactor, and two clarifier tanks were operated at ambient temperature (23°C). The sludge from an anaerobic digester in Kitakyushu, Japan was first enriched with QMA (60 g/L) and then approximately 4000 mg COD/L of enriched sludge was introduced into the reactor. As detailed parameters in a previous study [12], QMA (343 mg/L) was fed as the sole substrate continuously with a stepwise decrement in hydraulic retention time (HRT) to accommodate an increasing volumetric loading of 0.6 g C/L/d. Upon sampling at day 147 of reactor operation, the system displayed excellent performance in total organic carbon (TOC) removal efficiency (>90%). The full-scale UASB reactor in Tainan, Taiwan had a working volume of approximately 1000 m^3^ and has been operated for treating QMA-rich wastewater with HRT of 1.3–2 days without temperature control (18~26°C) for more than three years [4]. The influent mainly contained QMA with the concentrations of 455~1100 mg/L, which was equivalent to ~85% of total organic carbon and 98% of total organic nitrogen in wastewater. Sludge samples, UASB1a-UASB5a, from the full-scale UASB reactor were taken from the sampling ports located at heights of 30 cm, 100 cm, 200 cm, 300 cm, and 400 cm (Table 1). Upon sampling, the UASB reactor achieved a TOC removal efficiency of 85–90% with slightly high pH (~7.5) in the effluent and high methane content (>90%) in the biogas stream. From each sample, 20–40 mL of sludge was preserved at −80°C in a freezer until use. The concentrations of QMA were analyzed by an ion chromatograph DX-120 (Thermo Fisher, CA, USA).

### 2.2. Protein Extraction and In-Sol Digestion

Proteins were recovered from the sludge following the protocol of freeze-thaw and acetone precipitation described elsewhere [13]. The protein extracts (60 *μ*L) were first diluted with 50 mM ammonium bicarbonate and then reduced with 10 mM dithiothreitol at 25°C for 60 min, followed by cysteine-blocking with 40 mM iodoacetamide at 25°C for 30 min. Samples were digested with sequencing grade modified porcine trypsin (Promega, Madison, WI, USA) at 25°C for 16 hours. The peptides were then desalted and dried by vacuum centrifugation and store at −80°C until mass spectrometry analysis.

### 2.3. Online Two-Dimensional LC-MS/MS Analysis

The analysis of proteolytic peptides was performed on a nanoflow-HPLC system (Thermo Finnigan, Surveyor MS Pump Plus, Thermo Scientific) with a 2D linear ion trap mass spectrometer (LTQ-Orbitrap ELITE; Thermo Fisher, San Jose, CA, USA). Briefly, the desalted peptide mixtures were reconstituted in HPLC buffer A (30% acetonitrile/0.1% formic acid) and loaded onto a homemade column (Luna SCX 5 *μ*m, 0.5 × 100 mm). The peptides were then fractionated to 22 fractions by eluting with 0 to 100% HPLC buffer B (0.5 M ammonium chloride/30% acetonitrile/0.1% formic acid) using online 2D-HPLC (Dionex Ultimate 3000, Thermo Fisher). Each SCX fraction was diluted in-line prior to trap onto a reverse-phase column (Zorbax 300SB-C18, 0.3 ×5 mm; Agilent Technologies). The peptides were then separated on a HydroRP column (2.5 *μ*m, 75 *μ*m I.D. × 24 cm with a 15 *μ*m tip) using a multistep gradient of HPLC buffer C (99.9% acetonitrile/0.1% formic acid) for 65 minutes with a flow rate of 0.25 *μ*L/min.

The full-scan MS was performed in the Orbitrap over a range of m/z 350 to 2000 and a resolution of 60,000 at m/z 400. The 20 data-dependent MS/MS scan events were followed by one MS scan for the 20 most abundant precursor ions in the preview MS scan. The m/z values selected for MS/MS were dynamically excluded for 40 seconds with a relative mass window of 15 ppm. The electrospray voltage was set to 2.0 kV, and the temperature of the capillary was set to 200°C. MS and MS/MS automatic gain control were set to 1000 ms (full scan) and 200 ms (MS/MS) or 2 × 106 ions (full scan) and 3 × 103 ions (MS/MS) for maximum accumulated time or ions, respectively.

### 2.4. Protein Identification and Annotation

The mass spectrometry data analysis was carried out using Proteome Discoverer software (version 1.4, Thermo Fisher Scientific). The MS/MS spectra were searched against the UniProt database using the Mascot search engine (Matrix Science, London, UK; version 2.5). To detail the expressed function of predominant populations, specific genome sequences were searched separately. For peptide identification, 10 ppm mass tolerance was permitted for intact peptide masses, and 0.5 Da for CID fragment ions with allowance for one missed cleavage made from the trypsin digestion: oxidized methionine and acetyl (protein N-terminal) as variable modifications and carbamidomethyl (cysteine) as the fixed modifications. Peptide-spectrum matches were then filtered based on high confidence and Mascot search engine rank 1 of peptide identification to ensure an overall false discovery rate below 0.01. To ensure the protein identification, only proteins with at least two peptides identified in the LC-MS/MS analysis were included for analysis. The annotation of the identified proteins was performed with the updated Clusters of Orthologous Group (COG) (https://www.ncbi.nlm.nih.gov/COG/) and database of Kyoto Encyclopedia of Genes and Genomes (KEGG) (http://www.kegg.jp/kegg/).

### 2.5. DNA Recovery and Quantitative PCR

The samples were washed thrice with phosphate buffer (pH 7.4) and subjected to DNA recovery with a MoBio PowerSoil DNA isolation kit (Carlsbad, CA). The DNA quality was verified spectrophotometrically, and the concentrations of double-stranded DNA were determined with a PicoGreen quantitation reagent (Molecular Probes, Oregon, USA) in a TBS-380 Mini-Fluorometer (Turner BioSystem, CA, USA). An SYBR Green quantitative PCR (Q-PCR) was performed using a CFX96 real-time PCR detection system (BioRad, USA), with *bacteria*-specific primers (8F, 5′-AGA GTT TGA TCC TGG CTC AG-3′; 518R, 5′-GWA TTA CCG CGG CKG CTG-3′) and *archaea*-specific primers (915F, 5′-AGG AAT TGG CGG GGG AGC AC-3′; 1059R, 5′-GCC ATG CAC CWC CTC T-3′) [14, 15], respectively, to determine the quantities of bacterial and archaeal 16S rRNA genes in the sludge. The Q-PCR experiment was performed in triplicate, with each reaction solution (20 *μ*L) containing 200 nM of each primer, 10 *μ*L of 2X SYBR Green Supermix (BioRad), and 5–30 ng of the DNA template. The thermal program was set to 95°C for 3 min, followed by 45 cycles at 95°C for 5 s, 56°C for 30 s (for *bacteria*), or 54°C for 30 s (for *archaea*) and 72°C for 15 s; at this step of each cycle, the fluorescent products were monitored at a wavelength of 530 nm. The accuracy of the Q-PCR assay was confirmed through melting curve analysis and agarose gel electrophoresis. Calibration curves were obtained using 10-fold serial dilutions of known concentrations of cloned DNA samples that were prepared in the laboratory.

### 2.6. PCR Amplification, Clone Library Construction, and Phylogenetic Analysis of Archaeal 16S rRNA Genes

The archaeal communities obtained from various heights (UASB1a-UASB5a) in a methanogenic bioreactor were investigated by denaturing gradient gel electrophoresis- (DGGE-) cloning approaches through amplification of the 16S rRNA by using the primers Arc344f-mod and Arch958r-mod [16]. The amplicons were cloned to *Escherichia coli* DH5*α* cells using a pGEM-T easy vector (Promega, Madison, USA). The clones with the correct DNA inserts were screened through SP6/T7 PCR. Sequence types among the positive clones were detected by DGGE on a DCode System (BioRad). The DGGE profiles of the individual clones were compared with each other and with the DGGE profile of the entire community [17]; the profiles were obtained through seminested PCR amplification with the primers Arc344f-mod-GC and 524F-10-ext-rv (5′-TTA CCG CGG CTG RCA-3′) [16]. The sequences of the representative clones, referring to the phylotype, were obtained through the Sanger sequencing method and deposited in the GenBank database under the accession numbers KU569977–KU569987. The obtained sequences were compared with sequences in the GenBank database by using the BLAST search tool (http://blast.ncbi.nlm.nih.gov/Blast.cgi). A phylogenetic tree was constructed with the neighbor-joining method, and bootstrap resampling analysis was performed with 1000 replicates in the MEGA6 program [18].

### 2.7. 16S rRNA Gene High-Throughput Sequencing

The compositions of archaeal and bacterial populations were analyzed using a high-throughput sequencing method. The 16S rRNA genes of *archaea* were amplified using a barcoded fusion *archaea*-specific primer set (Arc344f-mod/Arch958r-mod) [16]. The PCR reaction mixtures comprised 10 ng of genomic DNA, 200 nM (each) of each primer, 0.2 mM dNTP (each), 1x FastStart Buffer, and 1.25 U of FastStart HiFi Polymerase (Roche, Indiana, USA) in a volume of 24 *μ*L. Reactions were conducted in a 9700 thermal cycler (Applied Biosystems, Foster, CA, USA) by denaturing DNA for 3 min at 94°C, followed by 35 cycles of 15 s at 94°C, 45 s at 52°C, and 1 min at 72°C, and a final extension at 72°C for 8 min. The PCR amplicons were purified using Agencourt AMPure XP Reagent (Beckman Coulter Inc., Beverly, Massachusetts, USA) and subsequently quantified using an Agilent Bioanalyzer (Agilent Technologies Inc., Deutschland, Waldbronn, Germany). Equimolar amounts of the PCR amplicons were mixed in a single tube, and pyrosequencing was performed using a 454 GS Junior, according to the manufacturer's instruction (Roche Applied Science, Branford, CT, USA).

For *bacteria*, the hypervariable region of the 16S rRNA gene was amplified using a barcoded fusion primer set comprising Pro341F and Pro805R [19]. The amplification solution (20 *μ*L) contained 10 *μ*L of 2X Phusion HF master mix (New England BioLabs, Ipswich, MA, USA), 0.5 *μ*M of each primer (with customized barcodes present on both primers for multiplex sequencing), and 50–150 ng of DNA template. The PCR conditions were as follows: initial denaturation at 95°C for 2 min, 30 cycles of annealing starting at 65°C (ending at 55°C) for 15 s, and extension at 68°C for 30 s. The amplicons in the triplicate samples were pooled and purified using an AMPureXP PCR Purification Kit (Agencourt, Brea, CA, USA) and quantified using a Qubit dsDNA HS Assay Kit on a Qubit 2.0 Fluorometer (Invitrogen, Carlsbad, CA, USA). To sequence the library preparation, Illumina adapters were attached to the amplicons by using an Illumina TruSeq DNA sample preparation kit, v2. Purified libraries were used for cluster generation and sequencing on the Illumina Miseq platform.

### 2.8. Analysis of High-Throughput Sequencing Data

Sequence reads were sorted on the basis of their respective barcodes into individual libraries. The sequences of primers, barcodes, and adaptors were then trimmed, and reads shorter than 150 bp and those containing ambiguous nucleotides were removed. The reverse sequences were complemented on the RDP Pipeline Initial Process. The qualified sequences were then aligned based on the RDP infernal and assigned phylogenetically using the RDP classifier at a 95% confidence level [20]. The cluster files were subsequently employed to generate rarefaction curves, which showed an accumulating trend among OTUs defined at the 97% similarity level relative to the total number of sequence reads. To normalize the uneven sequencing effects, the OTU table was 3X randomly rarefied subset of 12,000 and 20,000 sequences per sample for archaeal and bacterial libraries, respectively. The Pearson correlation between specific methanogen genera was obtained using Microsoft Excel 2010. The sequencing data were submitted to the EMBL-EBI European Nucleotide Archive under the study accession number PRJEB13976 and PRJEB14151.

## 3. Results

### 3.1. Microbial Diversity

The microbial communities of QMA-degrading sludge sample (CMJP) from a CMSS reactor and samples (UASB1a-UASB5a) from various heights in a full-scale UASB reactor were studied using tag-encoded amplicon sequencing of archaeal and bacterial 16S rRNA gene sequences. To assess the internal (within-sample) complexity of individual communities, various microbial diversity indices were compared. As shown in Table 1, the number of archaeal OTUs of UASB sludge displayed a cardinal distribution with the low values of 282–447 at the bottom and top of the reactor, and the highest value of up to 710 at 200 cm. According to an analysis of the Chao 1 index, species richness between the sludge samples collected at reactor heights of 100 cm and 400 cm was 1341 and 500, respectively, suggesting a 2.7-fold difference in the degree of complexity of archaeal diversity. The values of OTUs (218–299) and Chao 1 (270–384) for bacterial populations appeared to be lower than archaeal populations, even though a higher number of sequence reads was analyzed. The Shannon–Weaver index values of the samples taken at 100–300 cm were similar and were higher than the corresponding values of samples taken at heights of 30 and 400 cm. The values of Shannon–Weaver index for bacterial populations were significantly higher than archaeal populations (*t*-test, *p* = 0.01). The results showed that the archaeal populations displayed higher species richness but lower species diversity, as compared to the bacterial populations in the UASB reactor. Still, the species richness and diversity of archaeal populations and bacterial diversity in most samples of UASB reactor were higher than in the CMJP sample, which might be attributed to the influence of substrate complexity in real wastewater.

### 3.2. Microbial Community Structures

**Figure 1:**
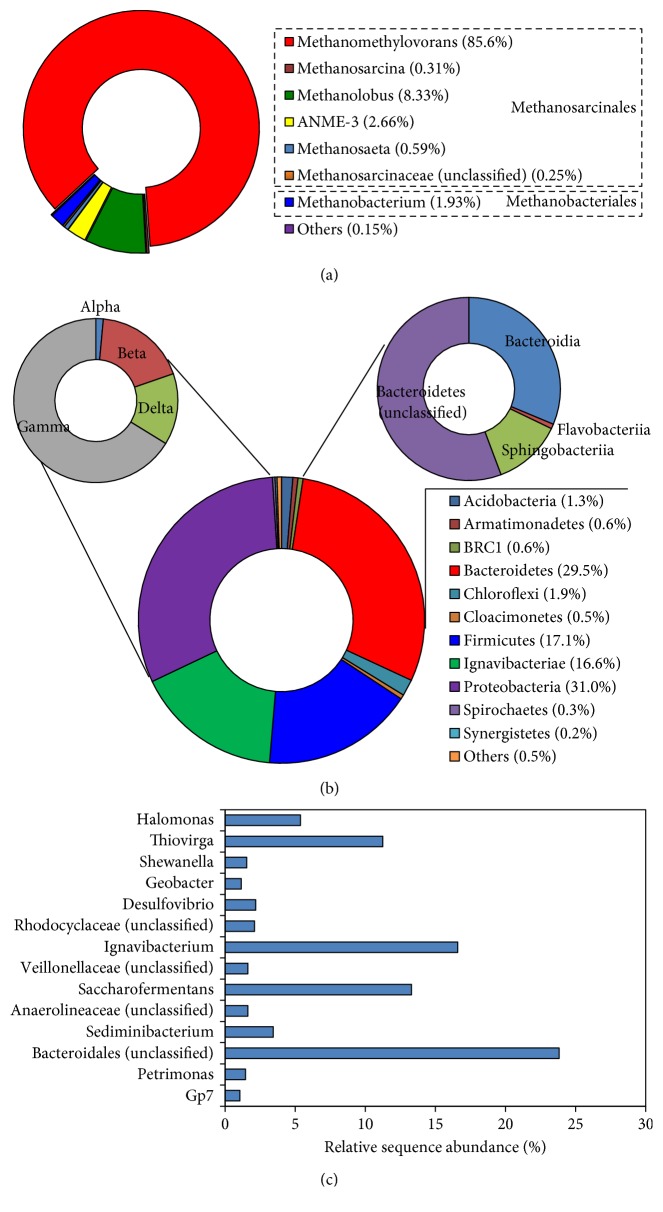
High-throughput sequencing analysis of archaeal and bacterial 16S rRNA sequences of sludge samples taken from a tetramethylammonium-degrading CMSS reactor. (a) Phylogenetic distribution of the detected archaeal populations. “Others” included the low abundant populations, namely, *Methanolinea* (0.01%), *Methanospirillum* (0.01%), *Methanobacteriaceae* (unclassified) (0.04%), and *Thermoplasmata* (0.09%). (b) Phylogenetic distribution of the detected bacterial populations at the phylum and class levels. “Others” included 10 bacterial phylum taxa with each abundance <0.05%. (c) Distribution of bacterial genera with relative sequence abundance >1%.

In the archaeal library of CMJP, more than 99.9% of the sequence reads were assigned to methanogenic *archaea* under *Euryarchaeota* and distributed with sequence abundance of 97.7, 2.2, and 0.04% in the order of *Methanosarcinales*, *Methanobacteriales*, and *Methanomicrobiales*, respectively. As shown in Figure 1(a), the members of the genera *Methanomethylovorans* (85.6%) and *Methanolobus* (8.3%) of the order *Methanosarcinales* account for 93.9% of the total reads, representing the most predominant types of methanogenic archaea in the CMSS reactor. A relatively low fraction of the sequences corresponded to acetotrophic and hydrogenotrophic methanogens, including the aceticlastic *Methanosaeta* (0.59%) and hydrogenotrophic methanogens such as *Methanobacterium* (1.93%), *Methanolinea* (0.01%), and *Methanospirillum* (0.01%). Accordingly, the results suggested that in the CMSS reactor, methane formation following the methylotrophic pathway was more critical than that following the acetotrophic and hydrogenotrophic pathways. Notably, approximately 2.7% of the total reads were assigned to the ANME-3 group, an archaeal population related phylogenetically to the order *Methanosarcinales* and thought to be capable of oxidizing methane to CO_2_ anaerobically [21]. Also, a low abundance of sequence reads (0.09%) was assigned to *Thermoplasmata*, whose members were recently recognized as methylotrophic methanogens [22].

The bacterial community structure in CMJP was relatively complex, as 25 bacterial phyla (reads > 0.01%) were detected. As shown in Figure 1(b), the phyla *Proteobacteria*, *Bacteroidetes*, *Firmicutes*, and *Ignavibacteriae* accounted for 31.0, 29.5, 17.1, and 16.6% of the total sequence reads, representing the top four predominant bacterial groups in the QMA-degrading consortium. Within the phyla, *Gammaproteobacteria* (accounting for 66.2% of *Proteobacteria*-related sequences) and the unclassified *Bacteroidetes* and *Bacteroidia* (accounting for 55.7 and 31.3% of *Bacteroidetes*-related sequences, resp.) were highly predominant.  Figure 1(c) shows a detailed analysis of the sequences at the genus level. The dominant bacterial genera with a sequence abundance of >5% included *Ignavibacterium* (16.6%), *Saccharofermentans* (13.3%), *Thiovirga* (11.2%), *Halomonas* (5.4%), and the unclassified *Bacteroidales* group (23.8%). Members of the genus *Thiovirga* are chemolithoautotrophic for sulfur oxidation [23], whereas the other three known genera are usually organotrophic. Their presence has been associated with the anaerobic fermentation of organic substances [24–27].

**Figure 2:**
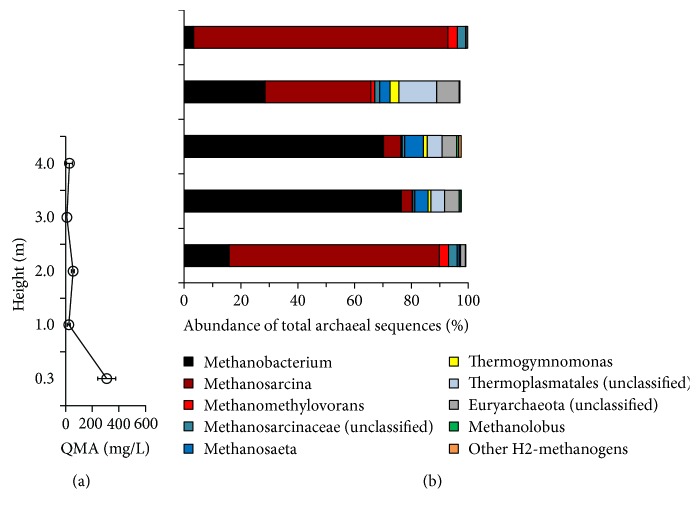
Tetramethylammonium (QMA) profile (a) and quantitative distribution of archaeal populations in the full-scale UASB reactor for treating tetramethylammonium-rich wastewater as revealed by the pyrosequencing 16S rRNA gene amplicons (sequence abundance >0.8%) (b). “Other H_2_ methanogens” included the low abundant populations, namely, *Methanolinea* (<0.07%), *Methanoculleus* (<0.01%), *Methanospirillum* (<0.03%), *Methanobrevibacter* (<0.03%), *Methanosphaera* (<0.01%), and *Methanococcus* (<0.01%).

As shown in Figure 2, the QMA profile and archaeal populations distributed distinctively in a full-scale UASB reactor. The decreasing trend of QMA concentrations with the reactor height revealed that the QMA was mostly degraded in the sludge bed (~1.0 m) (Figure 2(a)). In addition to the constantly low abundance of *Methanococcales*, the detected populations related to *Methanosarcinales*, *Methanobacteriales*, and *Thermoplasmatales* displayed a specific spatial distribution. To further elucidate the distribution, the genera data were analyzed in detail. As shown in Figure 2(b), at least three genera of methylotrophic methanogens were detected. Among them, the relative sequence abundance of *Methanomethylovorans* and *Methanolobus* in the samples remained low (<3.4%) in comparison with *Methanosarcina*. The relative sequence abundance of *Methanosarcina* varied markedly with the reactor height, being higher in sequence abundance at the bottom (74.0%) and top (89.5%) than at the middle (3.8–37.2%) of the USAB reactor. Notably, this spatial distribution of *Methanosarcina* appeared to exhibit an inverse relationship with aceticlastic *Methanosaeta* (0.08–6.6%) and hydrogenotrophic *Methanobacterium* (3.3–76.5%), with high Pearson's coefficients (*r*) of −0.961 and −0.968, respectively, but the distribution of *Methanosarcina* was associated positively with methylotrophic *Methanomethylovorans* (*r* = 0.992) (Figure S1). Except for *Methanobacterium*, all other detected hydrogenotrophic methanogens, including *Methanolinea*, *Methanoregula*, *Methanoculleus*, *Methanospirillum*, *Methanobrevibacter*, *Methanosphaera*, and *Methanococcus*, were present in relatively low abundance (approximately 0.77% in total). These results suggested that the archaeal populations exhibited a distinct spatial distribution in the UASB reactor, even though in which the upflow of inflow wastewater and gaseous products provided vertical mixing to the sludge.

The bacterial libraries of UASB sludge comprised totally 30 bacterial phyla (reads > 0.01%). As shown in Figure S2(a), the phyla *Proteobacteria* (12.4–58.6%), *Bacteroidetes* (15.8–27.1%), *Firmicutes* (15.2–19.4%), *Synergistetes* (2.6–8.4%), and *Caldiserica* (1.2–9.2%) represented the top five predominant bacterial groups in the sludge of UASB reactor. Figure S2(b) showed a detailed analysis of the abundant (>1%) sequence types at the genus level. Several sulfate-reducing bacteria such as *Sulfurospirillum*, and *Desulfovibrio*, homoacetogenic *Acetobacterium*, *Geobacter*, and *Anaerovorax* in the samples UASB1a (sampling height, 30 cm) and UASB5a (sampling height, 400 cm) shared similar sequence abundance higher than those in the sample UASB3a (sampling height, 200 cm). As shown in Figure S3, 11 bacterial phyla were shared by the UASB and CMSS sludge, while 14 and 9 bacterial phyla were specific to UASB and CMSS sludge, respectively. The difference of bacterial populations between the reactors might be partially attributed to the effects of influent substrates and lysed biomass.

### 3.3. Spatial Distribution of Archaeal Communities as Revealed by 16S rRNA Gene DGGE-Cloning

**Figure 3:**
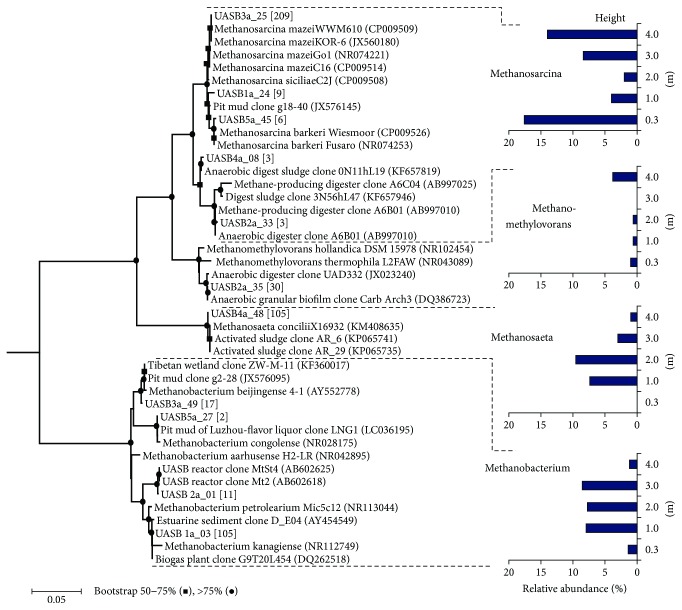
Phylogenetic tree and distribution of archaeal 16S rRNA genes retrieved from the sludge taken at various heights (0.3–4 m) of the full-scale UASB reactor for treating tetramethylammonium-rich wastewater. The 16S rRNA gene sequences were obtained through the DGGE-cloning approach, and the representative clones were shown in bold in the tree. The values in the square brackets indicated the numbers of clones that were similar to the representative phylotypes (>99% sequence similarity). The 16S rRNA gene sequence of *Methanopyrus kandleri* (NC003551) was used as an outgroup to root the tree. Bootstrap values of 50–75% and >75% were indicated with black circles and squares, respectively, at the nodes of the tree. The scale bar represented the estimated number of nucleotide changes per sequence position.

To validate the observed distinctive spatial distribution of archaeal populations, we employed the DGGE-cloning approach with the five samples from the UASB reactor. In total, 87 of 500 clones (100 clones with each sample) with 16S rRNA gene inserts with a unique banding profile were sequenced in the DGGE analysis and classified into 11 phylotypes within the phylum *Euryarchaeota*. As shown in Figure 3, most of these phylotypes were closely affiliated with four methanogen genera, namely, *Methanosarcina* (46% of total clones), *Methanobacterium* (27% of total clones), *Methanosaeta* (21% of total clones), and *Methanomethylovorans* (6% of total clones). The results from the DGGE-cloning analysis validated that all the three types of methanogen were predominant in the UASB reactor. The detected methanogens had a similar spatial distribution along the reactor height, as revealed by the pyrosequencing results. Figure S4 showed that *Methanosarcina* appeared to be more abundant (70−80% for bottom/top versus, 10−42% for middle) in the sludge samples taken at 0.3 m (bottom) and 4 m (top) of the UASB reactor, whereas the hydrogenotrophic *Methanobacterium* (6−7% for bottom/top versus 39−43% for middle) and the aceticlastic *Methanosaeta* (0−5% for bottom/top versus 15−48% for middle) populations were more abundant in the sludge samples taken at 1–3 m. As compared with other samples of UASB, the methylotrophic *Methanomethylovorans* was abundant at the top of the UASB reactor because 19% of total clones were detected in the UASB5a sample. Because of low number of clones screened, the DGGE-cloning analysis, however, did not detect some methanogens (e.g., *Methanolobus*) of low abundance, as suggested by pyrosequencing analysis. No sequence related to nonmethanogenic archaeon, *Thermogymnomonas*, and unclassified *Thermoplasmatales* was detected using DGGE-cloning analysis, but the relevant sequences could be retrieved using pyrosequencing. These dissimilarities are attributable to the biases associated with the PCR and cloning procedures, as well as sequencing depth [16, 28].

### 3.4. Quantitative Analysis of Archaeal and Bacterial Populations

**Figure 4:**
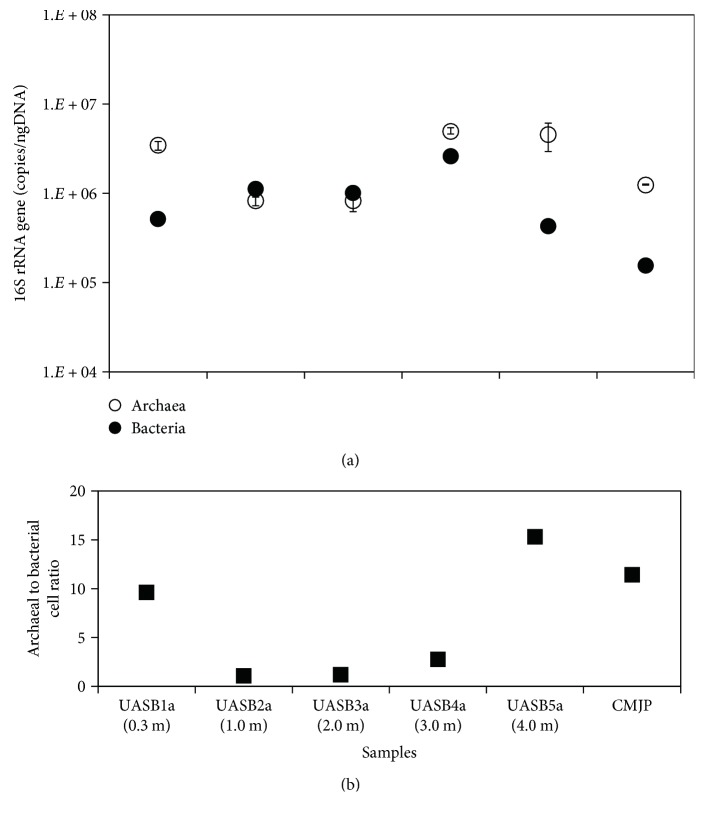
(a) 16S rRNA gene copies (on average) of archaeal and bacterial populations and (b) the estimated ratios of archaeal to bacterial populations in methanogenic sludge from tetramethylammonium-degrading CMSS (sample CMJP) and UASB reactors (samples UASB1a-UASB5a). Coefficients of variance (CV) for archaeal and bacterial Q-PCR experiments (triplicate analysis per sample) are 1.4~35.1% and 2.3~12.8%, respectively.

As revealed by Q-PCR, Figure 4(a) showed the distribution of archaeal and bacterial 16S rRNA gene copies of the sludge samples in this study. For samples UASB1a, UASB5a, and CMJP, the results showed that the 16S rRNA gene copies of archaeal populations (3.43~4.54 × 10^6^ copies/ng-DNA for UASB1a, and UASB5a; 1.23 × 10^6^ copies/ng-DNA for CMJP) were present in numerically higher proportions than in bacterial populations (4.31~5.18 × 10^5^ copies/ng-DNA; 1.56 × 10^5^ copies/ng-DNA). Because the copy numbers per archaeal and bacterial genome are, respectively, 2.5 and 3.6 on averages [29], the corresponding cell numbers can be estimated accordingly, which leads to an archaeal to bacterial cell ratio in the range of 9.6–15.3 (Figure 4(b)). For the sludge samples from the middle of UASB reactor, the archaeal and bacterial populations shared a similar abundance with a cell ratio estimated to be 1.1–1.2, which was lower than that in the bottom and top of the UASB reactor.

### 3.5. Proteomic Analysis of QMA-Degrading Consortium

Since the QMA as the sole substrate was applied to the reactor, the CMJP sample was very suitable for studying microbial activities associated with the QMA degradation using the proteomic analysis. Online two-dimensional proteomic analysis identified at least 748 protein entries with the prokaryotic protein database from UniProtKB/TrEMBL. The numbers of *archaea*-related proteins (706 proteins, 94.4% of total identified proteins) were much higher than the bacterial counterparts (42 proteins, 5.6% of total identified proteins). Among the *archaea*-related proteins, 667 proteins were related to 15 genera of cultivable archaeal methanogens. As shown in Figure S5(a), *M. hollandica* was the most dominant population that produced the highest number (523 proteins), followed by *Methanosarcina* (60 proteins), *Methanolobus* (29 proteins), uncultured archaeon (14 proteins), and *Methanococcoides* (12 proteins) in order. These top 5 abundant archaeal populations accounted for protein abundance up to 93.9% of total archaeal proteins detected. The detailed analysis of the archaeal proteins showed that a large number of proteins associated with methanogenesis of methylamines and methanol were produced by the methylotrophic methanogens, including *Methanosarcina*, *Methanolobus*, *Methanohalophilus*, and *Methanosalsum* (Table S1), and *M. hollandica* (Table S2). As shown in Figure S5(b), the number of identified proteins assigned to every bacterial species detected was relatively low (<3 proteins per species). The overall results of proteomic analysis were in well consistent with the community analysis data (Figure 1(a)) and suggested that the methylotrophic methanogens (especially *M. hollandica*) exhibited vigorous activities in CMJP sludge.

### 3.6. Protein Expression of *Methanomethylovorans hollandica*

**Figure 5:**
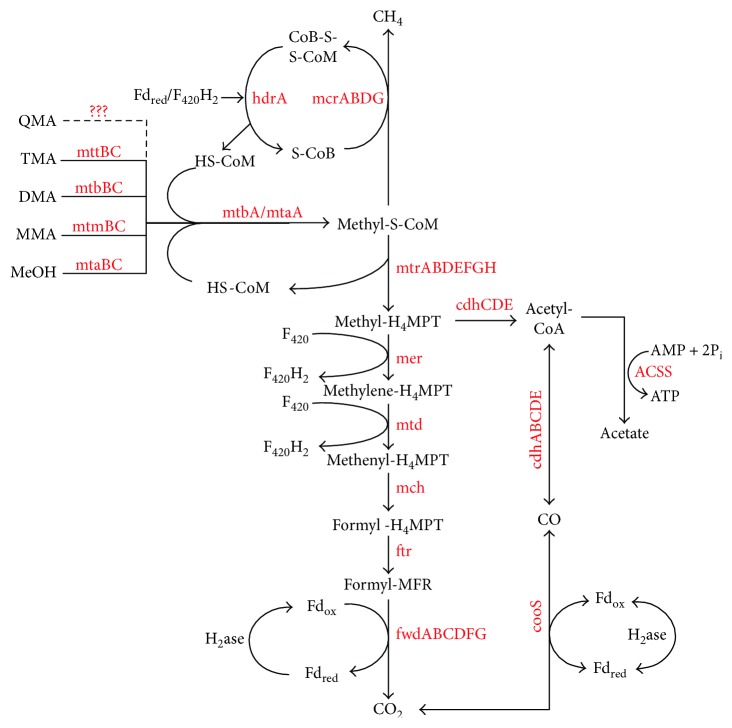
Proteins expressed in the pathways of methylotrophic, hydrogenotrophic, and acetotrophic methanogenesis of *Methanomethylovorans hollandica* in the conversion of tetramethylammonium (QMA) to methane. TMA: trimethylamine; DMA: dimethylamine; MMA: monomethylamine; MeOH: methanol; MFR: methanofuran; H_4_MPT: tetrahydromethanopterin; HS-CoM: coenzyme M; HS-CoB: coenzyme B; CoM-S-S-CoB: heterodisulfide of HS-CoM and HS-CoB; F_420_H_2_: reduced coenzyme F_420_; Fd_red_: reduced ferredoxin; Fd_ox_: oxidized ferredoxin; H_2_ase: hydrogenase. The proteins detected in the study were highlighted in red and referred to in Table S2.

The protein expression of *M. hollandica* was closely elaborated by matching the mass spectrometry data with the protein sequences coded by the *M. hollandica* genome. Approximately, 33.3% (841 genes) of coding genes predicted in the *M. hollandica* genome were converted to proteins. The annotation of the identified proteins with updated COG obtained 592 COGs in 21 categories and showed that the proteins with functions assigned to the categories J (translation and ribosomal structure and biogenesis), C (Energy conservation), E (amino acid transport and metabolism), and H (coenzyme transport and metabolism) were numerically abundant (Figure S6). It was observed that the proteins involved in various methanogenesis pathways were highly expressed (Figure 5, Table S2). The expression of abundant proteins mttBC/mtbA for trimethylamine, mtbBC/mtbA for dimethylamine, mtmBC/mtbA for monomethylamine, and mtaABC for methanol revealed the activities of *M. hollandica* in the transfer of methyl group from methanol and methylamines to form methyl-S-CoM, which was further reduced to methane by the methyl-CoM reductase (mcrABDG). The proteomic results showed all proteins (fwdABCDFG, ftr, mch, mtd, mer, and mtrABDEFGH) needed in the pathway of H_2_/CO_2_ methanogenesis, while the acetyl-CoA synthetase (ACSS) and acetyl-CoA decarbonylase/synthase complex (cdhABCDE) that participated in the pathway of acetotrophic methanogenesis were identified, too (Figure 5). In addition, the abundant proteins matched to ROS scavengers like superoxide dismutase (L0KZ58) and catalase (L0L0M1) of *Methanomethylovorans*, as that archaeon may be under oxidative stress in the reactor loaded with QMA. The detection of abundant NAD(P)H-nitrite reductase (L0KW74) and hydroxylamine reductase (L0KZ19 and L0KU78) and glutamate dehydrogenase (L0KYE3) and glutamine synthetase (L0KT19) (Table S2) suggested the activities of *M. hollandica* in the regulation of nitrogen metabolism.

## 4. Discussion

The overall results of this study revealed a central role of the *Methanosarcinales* in the conversion of QMA to methane in anaerobic reactors. To date, only marine methanogens have been obtained in pure cultures with QMA as the sole energy source. *Methanococcoides methylutens* was the first marine methanogen reported to grow with QMA [6–8]. This genus of marine methanogen, however, was not detected using PCR-based molecular methods but was detectable using a shotgun proteomic approach in the present study. The low abundance was likely due to its deteriorated growth in low-salt environments (<0.3–0.4 M sodium). Recently, *Methanolobus vulcani* obtained from brackish river sediment was also shown to grow with QMA in a wider range of salt concentrations (0.05–0.94 M sodium) [6]. In this study, the *Methanolobus* spp. could also inhabit the low-salt conditions, since the reactor was loaded with low sodium medium (approximately 10.4 mM).

The *Methanomethylovorans* populations are methylotrophic and frequently thrive in terrestrial (freshwater) ecosystems over a broad temperature range [30–35]. The proteomics data of this study suggested the methylotrophic activities of *M. hollandica* associated with QMA degradation (Figure 5). This result was in well accordance with our previous study [12] and suggested a demethylation of the QMA degradation by *M. hollandica*. However, the proteomic data did not uncover the protein entries for the demethylation of QMA. To search possible candidates, the amino acid sequences of proteins (MtqABC) that had been detected for the demethylation of QMA in *Methanococcoides* sp. [7] were matched to the sequence of *M. hollandica* genome. Three methyl-Co(III) methylamine-specific corrinoid protein: CoM methyltransferase (Metho_0037, Metho_0007, Metho_0355) of *M. hollandica* shared high-sequence similarity (69–74%) with the MtqA of *Methanococcoides* sp. but only one (mtbA, accession: L0KUG6/Metho_0007, Table S2) was detected in this study. This observation suggested that the *M. hollandica* used the same methyltransferase (mtbA) to mediate the formation of methyl-S-CoM for tetra and tri-, di-, and mono-methylamines (Figure 5). However, likely because of high protein-substrate specificity, the *M. hollandica* genome in the database did not contain any homolog to the MtqBC. To identify the corresponding genes/proteins responsible for QMA demethylation, the genome sequences of the exact degrader strains are needed accordingly.

As predicted with the KEGG pathway database, the *M. hollandica* genome possessed the gene sets involved in the methanogenesis pathways from acetate. Interestingly, our proteomic data revealed that the *M. hollandica* produced abundant acetyl-CoA synthetase (ACSS) and acetyl-CoA decarbonylase/synthase complex (cdhCDE) for the acetotrophic pathway. Because the *M. hollandica* is not aceticlastic [31], one possible explanation was that a fraction of methyl-S-CoM molecules might be converted to acetate through a reverse acetotrophic pathway. The acetate production was known to be produced only from CO and formate by *Methanosarcina acetivorans* [36]. Until recently, the homoacetogenesis (namely, acetate production from H_2_/CO_2_) was recognized in the new archaeal phylum *Bathyarchaeota*, in which some members were suggested to be capable of methylotrophic methanogenesis [37]. Alternatively, the observation might account for the autotrophic carbon assimilation (Figure 5), because the CO dehydrogenase (L0KWW2), which was the key enzyme in the reductive acetyl-CoA pathway (Wood-Ljungdahl pathway) was also expressed by *M. hollandica*. The trait of acetate production/assimilation would be beneficial to *M. hollandica* to conserve more energy in the metabolism of QMA for thriving in energy-limited ecosystems.

Unlike *Methanomethylovorans*, high species diversity of *Methanosarcina* was observed in both reactors. The sludge samples taken from the UASB reactor harbored at least five types of *Methanosarcina* 16S rRNA gene sequences. In the phylogenetic tree (Figure 3), the closest relative species, *M. mazei*, *M. siciliae*, and *M. barkeri*, have been reported to use methylamines for methane formation in pure cultures [38–41]. The proteomic analysis also detected the three and other three *Methanosarcina* species to function methylotrophically in the CMSS reactor. Our results showed that *Methanomethylovorans* and *Methanosarcina* outperformed other methylotrophic methanogens in dominating the CMSS system and the sludge bed of the UASB reactor, respectively. This suggests that the occurrence and dominance of methylotrophic methanogen populations could be influenced by the type of reactor and how it was operated. The methylotrophic *Methanosarcina* and *Methanomethylovorans* spp. compete with each other (depending on substrate utilization kinetics) and occupy the niches of high- and low-QMA concentrations, respectively [42]. Our findings were consistent with this argument. The CMSS system with its complete mixing conferred a consistently low QMA concentration (2–5 mg C/L), facilitating the dominance of the *Methanomethylovorans* population. In contrast to the uniformity with the CMSS reactor, the QMA profiles (48~162 mg C/L) in the UASB reactor, of which the flow regime was closer to a plug-flow type, were at higher concentrations at the inlet of the reactor than at higher levels, resulting in the formation of a favorable environment for *Methanosarcina* in the sludge bed. However, the concentration effects cannot account for the constantly low abundance of *Methanomethylovorans* and *Methanolobus* in the UASB reactor or the dominance of *Methanosarcina* in the zone near the outlet (height, 400 cm). In a previous study, quantitative analysis of samples taken from a UASB reactor on different dates yielded a similar distribution of methylotrophic methanogens but with higher abundance of *Methanomethylovorans* than *Methanosarcina* [35]. This suggested dynamic competition between *Methanomethylovorans* and *Methanosarcina*. The dominance of *Methanosarcina* in the zone (400 cm) close to reactor outlet might be somehow attributed to its high ability to resist oxidative stress in oxic environments [43].

Notably, the *Methanobacterium* spp. also dominated the UASB reactor, particularly in the center space (100–200 cm) (approximately 76.5% of total archaeal 16S rRNA sequence), suggesting that in addition to methylotrophic methanogenesis by *Methanosarcina*, a major fraction of methane in that zone could be formed through a hydrogenotrophic methanogenesis pathway. Interestingly, the percentage was extraordinarily higher than that of the hydrogenotrophic methanogens detected in the CMSS reactor (2.0%) and in the bottom and top of UASB reactor, as well as other anaerobic reactors degrading industrial wastewaters and sludge biomass (<45.6%) [35, 44]. This unusual was also related to the contrast of archaeal to bacterial cell ratios analyzed by using Q-PCR. However, the absolute abundance of *Methanobacterium* still remained low in the center of the UASB reactor (Figure 4). Reasonably, the H_2_ source to the growth of hydrogenotrophic *Methanobacterium* spp. could be attributed to the bacterial degradation of decayed sludge biomass and fermentable substrates in wastewater (~15% of TOC) [12].

Besides, the finding might be indicative of the methyl oxidation of methylamines (2) to H_2_ and CO_2_, which were converted to methane (3). Finke and coworkers (2007) proposed that the methylotrophic *Methanosarcina* can produce H_2_ through shifting the metabolism to oxidize more methyl groups of methylated substrates to CO_2_. The production and diffusive loss of H_2_ from the *Methanosarcina* cell could be achieved by growing with the hydrogen-scavenging methanogens to facilitate low H_2_ partial pressure conditions [11]. Consequently, the observed distribution of methanogens suggested a mutual interaction between methylotrophic *Methanosarcina* and hydrogen-scavenging *Methanobacterium* for the H_2_ transfer reaction to occur. Such interaction was not recognized in the CMSS reactor (dispersed cell growth) but was specific to the UASB reactor (attached cell growth) in this study and in the previous studies with marine sediment [45], methanol/acetate-degrading cocultures [46], and methanethiol-degrading granular sludge [34]. Considering absolute abundance of *Methanobacterium* and assumed conditions inside the UASB reactor (QMA = 1 mM, bicarbonate = 0.1 M, ammonium = 0.1 M, pH = 7, H_2_ partial pressure = 10^−5^ atm, and methane = 0.75 atm), the QMA degradation (2) became thermodynamically favorable (*Δ*G′ = −33.9 kJ/reaction). Since the degree of diverting the methyl oxidation into H_2_ production was dependent on H_2_ partial pressure [11], it is speculated that the syntrophic degradation of methylated compounds by *Methanosarcina* spp. could be facilitated at low H_2_ partial pressure conditions. The *Methanosarcina* spp. may be superior to *M. hollandica* to produce extracellular H_2_ during metabolizing methylated compounds, because the *Methanosarcina* genomes possessed the gene encoding membrane-bound hydrogenase [30, 47], but *M. hollandica* lacked the kind of genes in the genome. *Methanosarcina* spp. have the potential to use the hydrogenotrophic pathway for methane production. However, whether simultaneous methylotrophic and hydrogenotrophic activities could occur in the same organism remains unclear, and further study is required to confirm this.

Aceticlastic *Methanosaeta* with low-medium sequence abundance (4.7–6.6%, Figure 2(b)) inhabited the zone (1-2 m height in UASB reactor) where methane was largely produced from H_2_/CO_2_. The growth of *Methanosaeta* in the CMSS and UASB reactors might not have been inhibited by ammonia toxicity because the ammonia concentrations (approximately 15–54 mM) resulting from the degradation of QMA were markedly lower than the reported inhibitory levels (>75 mM) [48, 49]. In the UASB reactor, the acetate sources could be partially attributable to fermentation of the decay of sludge biomass and substrates in sanitary wastewater, as well as the potential acetogenesis of methylotrophic methanogens. It was also likely the contribution from the bacterial homoacetogenesis. This was further supported by the results of the high-throughput analysis of bacterial 16S rRNA genes, which showed detectable abundance of homoacetogens such as *Acetobacterium* (1.0–4.4%) and *Clostridium* (0.05–1.48%) (Figure S2(b)). These observations may in part account for higher relative abundance of *Methanosaeta* in the UASB reactor (~6.61%) than the CMSS reactor (0.59%). Since the activity of homoacetogenic populations in the conversion of methylated compounds required close association with hydrogenotrophic methanogens [34], the nexus of actual microbial interactions and metabolisms in the UASB reactor was far complicated and should be further studied in the future.

## 5. Conclusions

In the present study, the microbial community structures of methanogenic sludge samples obtained from QMA-degrading CMSS and UASB reactors were contrasted. The overall results showed high degree of diversity of freshwater methylotrophic methanogens to sustain the anaerobic degradation of QMA to methane. In particular, the methylotrophic *Methanomethylovorans* sp. highly dominated in the CMSS reactor, whereas diverse methylotrophic *Methanosarcina* spp. possibly in the association with hydrogenotrophic *Methanobacterium* distributed abundantly in the UASB reactor, which suggested the effects of the reactor configuration and operation on the QMA-degrading communities. This finding advances the understanding of methanogenic degradation of quaternary amines in the engineering environments and may facilitate improving reactor technology used in the anaerobic treatment of relevant wastewater.

## Figures and Tables

**Table 1:** Reads and diversity indices obtained from the high-throughput analysis of archaeal and bacterial 16S rRNA gene amplicons for sludge samples taken from tetramethylammonium-degrading CMSS and UASB reactors.

Sample ID	Reactor	Target	Height (cm)	Reads^1^	Richness^2^	Chao1^3^	Shannon^4^
UASB1a	UASB	*Archaea*	30	12,000	447	839	3.30
UASB2a	UASB	*Archaea*	100	12,000	703	1341	3.94
UASB3a	UASB	*Archaea*	200	12,000	710	1174	4.13
UASB4a	UASB	*Archaea*	300	12,000	641	1196	4.09
UASB5a	UASB	*Archaea*	400	12,000	282	500	2.04
UASB1a	UASB	*Bacteria*	30	20,000	218	270	5.02
UASB3a	UASB	*Bacteria*	200	20,000	249	266	5.88
UASB5a	UASB	*Bacteria*	400	20,000	299	384	4.37
CMJP	CMSS	*Archaea*	NA	12,000	250	367	2.75
CMJP	CMSS	*Bacteria*	NA	20,000	232	375	2.54

^1^Number of qualified 16S rRNA sequence reads. ^2^Number of detected OTUs (97% similarity). ^3^Chao1 richness estimate; a higher number represents a higher degree of diversity. ^4^Shannon–Weiner index; a higher number represents a higher degree of diversity. NA: not applicable.

## Supplementary Materials

Supplementary Materials Table S1: annotation of proteins related to the conversion of methylamines by the methanogens in the CMJP sample analyzed in this study. Table S2: annotation of proteins related to the conversion of methylamines, methanogenesis, and nitrogen metabolism in *Methanomethylovorans hollandica* detected in the CMJP sample in this study. Figure S1: (a) correlation of *Methanosarcina* versus *Methanosaeta* and *Methanomethylovorans*. (b) Correlation of *Methanosarcina* versus *Methanobacterium*. The lowercase “r” denotes the Pearson coefficient. Figure S2: high-throughput sequencing analysis of bacterial 16S rRNA sequences of sludge samples taken from different heights (30 cm, 200 cm, and 400 cm) of a full-scale UASB reactor treating tetramethylammonium-rich wastewater. Figure S3: Venn diagram analysis of bacterial phyla detected with CMJP and UASB samples. Figure S4: quantitative distribution of archaeal populations in the full-scale UASB reactor for treating tetramethylammonium-rich wastewater as revealed by a DGGE-cloning approach. Figure S5: numerical distribution and accumulated relative abundance of the identified proteins from (a) archaeal and (b) bacterial members in the sludge sampled from a tetramethylammonium-degrading CMSS reactor. Figure S6: distribution of the identified proteins assigned to the functional categories based on cluster of orthologous group (COG) classification.

## References

[1] Hu T. H., Whang L. M., Liu P. W. (2012). Biological treatment of TMAH (tetra-methyl ammonium hydroxide) in a full-scale TFT-LCD wastewater treatment plant. *Bioresource Technology*.

[2] Lin H. L., Chen S. K., Huang Y. W., Chen W. C., Chien W. C., Cheng S. S. (2016). A combined upflow anaerobic sludge bed, aerobic, and anoxic fixed-bed reactor system for removing tetramethylammonium hydroxide and nitrogen from light-emitting diode wastewater. *Environmental Technology*.

[3] Lin H.-L., Chen B.-K., Hsia H.-P. (2011). Use of two-stage biological process in treating thin film transistor liquid crystal display wastewater of tetramethylammonium hydroxide. *Sustainable Environment Research*.

[4] Chang K.-F., Yang S.-Y., You H.-S., Pan J. R. (2008). Anaerobic treatment of tetramethylammonium hydroxide (TMAH) containing wastewater. *IEEE Transactions on Semiconductor Manufacturing*.

[5] Krzycki J. A. (2004). Function of genetically encoded pyrrolysine in corrinoid-dependent methylamine methyltransferases. *Current Opinion in Chemical Biology*.

[6] Ticak T., Hariraju D., Arcelay M. B., Arivett B. A., Fiester S. E., Ferguson D. J. (2015). Isolation and characterization of a tetramethylammonium-degrading Methanococcoides strain and a novel glycine betaine-utilizing Methanolobus strain. *Archives of Microbiology*.

[7] Asakawa S., Sauer K., Liesack W., Thauer R. K. (1998). Tetramethylammonium: coenzyme M methyltransferase system from Methanococcoides sp. *Archives of Microbiology*.

[8] Tanaka K. (1994). Anaerobic degradation of tetramethylammonium by a newly isolated marine methanogen. *Journal of Fermentation and Bioengineering*.

[9] King G. M. (1984). Utilization of hydrogen, acetate, and “noncompetitive”; substrates by methanogenic bacteria in marine sediments. *Geomicrobiology Journal*.

[10] Weijma J., Stams A. J. (2001). Methanol conversion in high-rate anaerobic reactors. *Water Science and Technology*.

[11] Finke N., Hoehler T. M., Jorgensen B. B. (2007). Hydrogen ‘leakage’ during methanogenesis from methanol and methylamine: implications for anaerobic carbon degradation pathways in aquatic sediments. *Environmental Microbiology*.

[12] Liu B., Yoshinaga K., Wu J.-H. (2016). Kinetic analysis of biological degradation for tetramethylammonium hydroxide (TMAH) in the anaerobic activated sludge system at ambient temperature. *Biochemical Engineering Journal*.

[13] Huang H.-J., Chen W.-Y., Wu J.-H. (2014). Total protein extraction for metaproteomics analysis of methane producing biofilm: the effects of detergents. *International Journal of Molecular Sciences*.

[14] Haakensen M., Dobson C., Deneer H., Ziola B. (2008). Real-time PCR detection of bacteria belonging to the Firmicutes phylum. *International Journal of Food Microbiology*.

[15] Ji Y., Kim H., Park H. (2012). Modulation of the murine microbiome with a concomitant anti-obesity effect by Lactobacillus rhamnosus GG and Lactobacillus sakei NR28. *Beneficial Microbes*.

[16] Pires A. C., Cleary D. F., Almeida A. (2012). Denaturing gradient gel electrophoresis and barcoded pyrosequencing reveal unprecedented archaeal diversity in mangrove sediment and rhizosphere samples. *Applied and Environmental Microbiology*.

[17] Puskarova A., Buckova M., Habalova B., Krakova L., Makova A., Pangallo D. (2016). Microbial communities affecting albumen photography heritage: a methodological survey. *Scientific Reports*.

[18] Tamura K., Stecher G., Peterson D., Filipski A., Kumar S. (2013). MEGA6: molecular evolutionary genetics analysis version 6.0. *Molecular Biology and Evolution*.

[19] Takahashi S., Tomita J., Nishioka K., Hisada T., Nishijima M. (2014). Development of a prokaryotic universal primer for simultaneous analysis of bacteria and archaea using next-generation sequencing. *PLoS One*.

[20] Wang Q., Garrity G. M., Tiedje J. M., Cole J. R. (2007). Naive Bayesian classifier for rapid assignment of rRNA sequences into the new bacterial taxonomy. *Applied and Environmental Microbiology*.

[21] Scheller S., Goenrich M., Boecher R., Thauer R. K., Jaun B. (2010). The key nickel enzyme of methanogenesis catalyses the anaerobic oxidation of methane. *Nature*.

[22] Poulsen M., Schwab C., Jensen B. B. (2013). Methylotrophic methanogenic Thermoplasmata implicated in reduced methane emissions from bovine rumen. *Nature Communications*.

[23] Ito T., Sugita K., Yumoto I., Nodasaka Y., Okabe S. (2005). Thiovirga sulfuroxydans gen. nov., sp. nov., a chemolithoautotrophic sulfur-oxidizing bacterium isolated from a microaerobic waste-water biofilm. *International Journal of Systematic and Evolutionary Microbiology*.

[24] Chen S., Niu L., Zhang Y. (2010). Saccharofermentans acetigenes gen. nov., sp. nov., an anaerobic bacterium isolated from sludge treating brewery wastewater. *International Journal of Systematic and Evolutionary Microbiology*.

[25] Mata J. A., Martinez-Canovas J., Quesada E., Bejar V. (2002). A detailed phenotypic characterisation of the type strains of Halomonas species. *Systematic and Applied Microbiology*.

[26] Iino T., Mori K., Uchino Y., Nakagawa T., Harayama S., Suzuki K. (2010). Ignavibacterium album gen. nov., sp. nov., a moderately thermophilic anaerobic bacterium isolated from microbial mats at a terrestrial hot spring and proposal of Ignavibacteria classis nov., for a novel lineage at the periphery of green sulfur bacteria. *International Journal of Systematic and Evolutionary Microbiology*.

[27] Liu Z., Frigaard N. U., Vogl K. (2012). Complete genome of Ignavibacterium album, a metabolically versatile, flagellated, facultative anaerobe from the phylum Chlorobi. *Frontiers in Microbiology*.

[28] Forney L. J., Zhou X., Brown C. J. (2004). Molecular microbial ecology: land of the one-eyed king. *Current Opinion in Microbiology*.

[29] Stoddard S. F., Smith B. J., Hein R., Roller B. R., Schmidt T. M. (2015). rrnDB: improved tools for interpreting rRNA gene abundance in bacteria and archaea and a new foundation for future development. *Nucleic Acids Research*.

[30] Liu Y., Whitman W. B. (2008). Metabolic, phylogenetic, and ecological diversity of the methanogenic archaea. *Annals of the New York Academy of Sciences*.

[31] Lomans B. P., Maas R., Luderer R. (1999). Isolation and characterization of Methanomethylovorans hollandica gen. nov., sp. nov., isolated from freshwater sediment, a methylotrophic methanogen able to grow on dimethyl sulfide and methanethiol. *Applied and Environmental Microbiology*.

[32] Roest K., Altinbas M., Paulo P. L. (2005). Enrichment and detection of microorganisms involved in direct and indirect methanogenesis from methanol in an anaerobic thermophilic bioreactor. *Microbial Ecology*.

[33] Jiang B., Parshina S. N., van Doesburg W., Lomans B. P., Stams A. J. (2005). Methanomethylovorans thermophila sp. nov., a thermophilic, methylotrophic methanogen from an anaerobic reactor fed with methanol. *International Journal of Systematic and Evolutionary Microbiology*.

[34] de Bok F. A., van Leerdam R. C., Lomans B. P. (2006). Degradation of methanethiol by methylotrophic methanogenic archaea in a lab-scale upflow anaerobic sludge blanket reactor. *Applied and Environmental Microbiology*.

[35] Wu J. H., Chuang H. P., Hsu M. H., Chen W. Y. (2013). Use of a hierarchical oligonucleotide primer extension approach for multiplexed relative abundance analysis of methanogens in anaerobic digestion systems. *Applied and Environmental Microbiology*.

[36] Rother M., Metcalf W. W. (2004). Anaerobic growth of Methanosarcina acetivorans C2A on carbon monoxide: an unusual way of life for a methanogenic archaeon. *Proceedings of the National Academy of Sciences of the United States of America*.

[37] He Y., Li M., Perumal V. (2016). Genomic and enzymatic evidence for acetogenesis among multiple lineages of the archaeal phylum Bathyarchaeota widespread in marine sediments. *Nature Microbiology*.

[38] Hippe H., Caspari D., Fiebig K., Gottschalk G. (1979). Utilization of trimethylamine and other N-methyl compounds for growth and methane formation by Methanosarcina barkeri. *Proceedings of the National Academy of Sciences of the United States of America*.

[39] Sowers K. R., Baron S. F., Ferry J. G. (1984). Methanosarcina acetivorans sp. nov., an acetotrophic methane-producing bacterium isolated from marine sediments. *Applied and Environmental Microbiology*.

[40] Ni S., Woese C. R., Aldrich H. C., Boone D. R. (1994). Transfer of Methanolobus siciliae to the genus Methanosarcina, naming it Methanosarcina siciliae, and emendation of the genus Methanosarcina. *International Journal of Systematic Bacteriology*.

[41] Veit K., Ehlers C., Schmitz R. A. (2005). Effects of nitrogen and carbon sources on transcription of soluble methyltransferases in Methanosarcina mazei strain Go1. *Journal of Bacteriology*.

[42] Whang L. M., Hu T. H., Liu P. W. (2015). Molecular analysis of methanogens involved in methanogenic degradation of tetramethylammonium hydroxide in full-scale bioreactors. *Applied Microbiology and Biotechnology*.

[43] Angel R., Matthies D., Conrad R. (2011). Activation of methanogenesis in arid biological soil crusts despite the presence of oxygen. *PLoS One*.

[44] Narihiro T., Terada T., Ohashi A. (2009). Quantitative detection of culturable methanogenic archaea abundance in anaerobic treatment systems using the sequence-specific rRNA cleavage method. *The ISME Journal*.

[45] King G. M., Klug M. J., Lovley D. R. (1983). Metabolism of acetate, methanol, and methylated amines in intertidal sediments of lowes cove, maine. *Applied and Environmental Microbiology*.

[46] Phelps T. J., Conrad R., Zeikus J. G. (1985). Sulfate-dependent interspecies H_2_ transfer between Methanosarcina barkeri and Desulfovibrio vulgaris during coculture metabolism of acetate or methanol. *Applied and Environmental Microbiology*.

[47] Thauer R. K., Kaster A. K., Seedorf H., Buckel W., Hedderich R. (2008). Methanogenic archaea: ecologically relevant differences in energy conservation. *Nature Reviews. Microbiology*.

[48] Westerholm M., Leven L., Schnurer A. (2012). Bioaugmentation of syntrophic acetate-oxidizing culture in biogas reactors exposed to increasing levels of ammonia. *Applied and Environmental Microbiology*.

[49] Werner J. J., Garcia M. L., Perkins S. D. (2014). Microbial community dynamics and stability during an ammonia-induced shift to syntrophic acetate oxidation. *Applied and Environmental Microbiology*.

