# Supplementary material for: Community and Proteomic Analysis of Anaerobic Consortia Converting Tetramethylammonium to Methane
Source: Archaea. 2017 Dec 17;2017:2170535. doi: 10.1155/2017/2170535 (PMC5748143; doi:10.1155/2017/2170535)
Supplement: Supplementary Materials — Table S1: annotation of proteins related to the conversion of methylamines by the methanogens in the CMJP sample analyzed in this study. Table S2: annotation of proteins related to the conversion of methylamines, methanogenesis, and nitrogen metabolism in Methanomethylovorans hollandica detected in the CMJP sample in this study. Figure S1: (a) correlation of Methanosarcina versus Methanosaeta and Methanomethylovorans. (b) Correlation of Methanosarcina versus Methanobacterium. The lowercase “r” denotes the Pearson coefficient. Figure S2: high-throughput sequencing analysis of bacterial 16S rRNA sequences of sludge samples taken from different heights (30 cm, 200 cm, and 400 cm) of a full-scale UASB reactor treating tetramethylammonium-rich wastewater. Figure S3: Venn diagram analysis of bacterial phyla detected with CMJP and UASB samples. Figure S4: quantitative distribution of archaeal populations in the full-scale UASB reactor for treating tetramethylammonium-rich wastewater as revealed by a DGGE-cloning approach. Figure S5: numerical distribution and accumulated relative abundance of the identified proteins from (a) archaeal and (b) bacterial members in the sludge sampled from a tetramethylammonium-degrading CMSS reactor. Figure S6: distribution of the identified proteins assigned to the functional categories based on cluster of orthologous group (COG) classification. [file 2170535.f1.pdf]

Supporting Information for Manuscript Submitted to *Archaea*

Community and Proteomic Analysis of Anaerobic Consortia Converting Tetramethylammonium to Methane

Wei-Yu Chen[1], Lucia Kraková[2], Jer-Horng Wu[1]*, Domenico Pangallo[2], Lenka Jeszeová[2], Bing Liu[3], Hidenari Yasui[3]

,

[1]Department of Environmental Engineering, National Cheng Kung University, No.1, University Road, East District, Tainan City 701, Taiwan (ROC)
[2]Institute of Molecular Biology, Slovak Academy of Sciences, Dubravska cesta 21, 84551 Bratislava, Slovakia
[3]Faculty of Environmental Engineering, The University of Kitakyushu, 1-1, Hibikino, Wakamatsu, Kitakyushu, Japan 808-0135

Table S1 Annotation of proteins related to the conversion of methylamines by the methanogens in the CMJP sample analyzed in this study.

Accession	Organism	gene	Annotation	Coverage (%)	Peptides (#)	Relative abundance (%)
A0A0E3SIM3	Methanosarcina barkeri 3	MSBR3_0618	Dimethylamine:corrinoid methyltransferase	17.13	21	1.39
A0A0E3Q5F5	Methanosarcina vacuolata Z-761	MSVAZ_1366	Methanol methyltransferase corrinoid protein	12.94	15	0.28
A0A0E3P0I6	Methanosarcina siciliae T4/M	MSSIT_0113	Monomethylamine:corrinoid methyltransferase	23.58	20	0.48
A0A0E3WTL8	Methanosarcina lacustris Z-7289	MSLAZ_3204	Monomethylamine:corrinoid methyltransferase	15.94	9	0.24
A0A0E3KRC1	Methanosarcina thermophila CHTI-55	MSTHC_1357	Monomethylamine:corrinoid methyltransferase	18.12	10	0.22
A0A0E3WT78	Methanosarcina lacustris Z-7289	MSLAZ_2479	Dimethylamine methyltransferase corrinoid protein	25.00	11	0.13
A0A0E3Q3C8	Methanosarcina vacuolata Z-761	MSVAZ_0479	Trimethylamine methyltransferase corrinoid protein	17.05	6	0.09
A0A0E3RQP4	Methanosarcina mazei LYC	MSMAL_2486	Trimethylamine:corrinoid methyltransferase / pyrrolysine-containing	18.32	10	0.10
A0A0E3Q2X7	Methanosarcina vacuolata Z-761	MSVAZ_0478	Trimethylamine:corrinoid methyltransferase	11.92	7	0.08
A0A0E3P3C2	Methanosarcina siciliae T4/M	MSSIT_1254	Methanol:corrinoid methyltransferase	5.64	5	0.07
A0A0G3CI53	Methanosarcina barkeri CM1	MCM1_3452	Methylcobalamin:coenzyme M methyltransferase	9.14	4	0.04
A0A0P0IVU9	Methanosarcina sp. 795	AAY43_03680	Monomethylamine corrinoid protein 1	10.60	3	0.02
A0A1E7GC69	Methanosarcina sp. Ant1	BGV40_08935	Dimethylamine corrinoid protein 3	13.43	7	0.10
A0A0F8D0L8	Methanosarcina sp. 2.H.T.1A.6	EO98_01420	Dimethylamine corrinoid protein 3	11.11	8	0.12
A0A1B8X0V8	Methanohalophilus sp. DAL1	A9957_09265	Trimethylamine:corrinoid methyltransferase	6.91	5	0.07
K4MEZ0	Methanolobus psychrophilus R15	Mpsy_1677	Monomethylamine corrinoid methyltransferase	15.92	17	0.45
W9DRF0	Methanolobus tindarius DSM 2278	MettiDRAFT_1545	Methyltransferase cognate corrinoid protein	24.40	11	0.13
K4MCC1	Methanolobus psychrophilus R15	Mpsy_0908	Methanol corrinoid protein	13.83	7	0.14
A0A139CSW5	Methanolobus sp. T82-4	AWU59_702	Methanol corrinoid methyltransferase	6.07	5	0.07
F7XQH1	Methanosalsum zhilinae	Mzhil_0605	Dimethylamine---corrinoid protein Co-methyltransferase	16.52	13	1.27
F7XPR5	Methanosalsum zhilinae	Mzhil_0461	Methyltransferase cognate corrinoid protein	17.59	12	0.19
F7XPR4	Methanosalsum zhilinae	Mzhil_0460	Trimethylamine methyltransferase	9.59	2	0.01

Table S2 Annotation of proteins related to the conversion of methylamines, methanogenesis and nitrogen metabolism in *Methanomethylovorans hollandica* detected in the CMJP sample in this study.

Accession	gene	Annotation	Coverage (%)	Peptides (#)	Relative abundance (%)	Pathway
L0KUI1	mttB	Trimethylamine:corrinoid methyltransferase	59.48	114	1.87	Methylotrophic
L0KT96	mttC	Trimethylamine corrinoid protein	27.44	17	0.16	Methylotrophic
L0L090	mttC	Trimethylamine corrinoid protein	14.35	4	0.03	Methylotrophic
L0KTA2	mtbB	Dimethylamine:corrinoid methyltransferase	60.52	100	2.93	Methylotrophic
L0KX52	mtbC	Dimethylamine corrinoid protein	51.40	34	0.39	Methylotrophic
L0KUG6	mtbA	methyl-Co(III) methylamine-specific corrinoid protein]:coenzyme M methyltransferase	28.61	20	0.19	Methylotrophic
L0KT85	mtmB	Monomethylamine:corrinoid methyltransferase	51.75	90	2.54	Methylotrophic
L0KSD3	mtmC	Monomethylamine corrinoid protein	39.35	25	0.23	Methylotrophic
L0KWJ7	mtmB	Monomethylamine:corrinoid methyltransferase	8.32	3	0.01	Methylotrophic
L0KWE8	mtaA	Methyltransferase, MtaA/CmuA family(methanol-specific corrinoid protein)	43.07	56	0.93	Methylotrophic
L0KY68	mtaC	Methanol corrinoid protein	48.62	31	0.36	Methylotrophic
L0KYG3	mtaB	Methanol-cobalamin methyltransferase B subunit	36.07	37	0.29	Methylotrophic
L0KYQ5	mtaC	Methanol corrinoid protein	5.91	2	0.01	Methylotrophic
L0KYC2	fwdA	Formylmethanofuran dehydrogenase subunit A	55.14	79	0.76	CO2 reduction
L0KWB0	fwdC	Formylmethanofuran dehydrogenase subunit C	50.19	43	0.45	CO2 reduction
L0KU81	fwdB	Formylmethanofuran dehydrogenase subunit B	31.72	37	0.40	CO2 reduction
L0KY21	fwdF	NADH:ubiquinone oxidoreductase chain I-like protein	47.06	32	0.30	CO2 reduction
L0KV15	fwdD	Formylmethanofuran dehydrogenase subunit D	44.70	13	0.09	CO2 reduction
L0KX24	fwdB	Formylmethanofuran dehydrogenase subunit B	22.71	14	0.09	CO2 reduction
L0KXC2	fwdF	NADH:ubiquinone oxidoreductase chain I-like protein	9.13	6	0.04	CO2 reduction
L0KXZ1	fwdD	Formylmethanofuran dehydrogenase subunit D	14.18	2	0.01	CO2 reduction
L0L0T0	fwdG	4Fe-4S ferredoxin	11.18	2	0.01	CO2 reduction
L0L2E9	ftr	Formylmethanofuran--tetrahydromethanopterin formyltransferase	44.30	56	0.86	CO2 reduction
L0KYG6	mch	Methenyltetrahydromethanopterin cyclohydrolase	42.50	20	0.21	CO2 reduction
L0KTR5	mtd	F420-dependent methylenetetrahydromethanopterin dehydrogenase	38.69	18	0.15	CO2 reduction
L0L1X7	mer	5,10-methylenetetrahydromethanopterin reductase	52.76	38	0.41	CO2 reduction

L0L001	mtrH	N5-methyltetrahydromethanopterin:coenzyme M methyltransferase subunit H	34.59	34	0.56	CO2 reduction
L0L1V0	mtrB	Tetrahydromethanopterin S-methyltransferase subunit B	28.97	13	0.12	CO2 reduction
L0L1I6	mtrA	Tetrahydromethanopterin S-methyltransferase subunit A	47.30	23	0.19	CO2 reduction
L0KYS5	mtrG	Tetrahydromethanopterin S-methyltransferase subunit G	65.79	11	0.07	CO2 reduction
L0KXS5	mtrE	Tetrahydromethanopterin S-methyltransferase subunit E	22.59	11	0.06	CO2 reduction
L0KXS0	mtrF	Tetrahydromethanopterin S-methyltransferase subunit F	33.77	3	0.03	CO2 reduction
L0KYT1	mtrD	Tetrahydromethanopterin S-methyltransferase subunit D	10.80	4	0.02	CO2 reduction
L0L1E2	ACSS	Acetyl-CoA synthetase	9.53	10	0.06	Acetotrophic
L0KWP3	ACSS	Acetyl-CoA synthetase	5.02	4	0.02	Acetotrophic
L0L0Q6	ACSS	Acetyl-CoA synthetase	2.39	2	0.01	Acetotrophic
L0KVQ7	cdhC	Acetyl-CoA decarbonylase/synthase complex subunit beta	42.86	39	0.31	Acetotrophic
L0KYY5	cdhA	Acetyl-CoA decarbonylase/synthase complex subunit alpha	36.07	56	0.58	Acetotrophic
L0KWZ4	cdhB	Acetyl-CoA decarbonylase/synthase complex subunit epsilon	17.16	18	0.19	Acetotrophic
L0KYR3	cdhD	Acetyl-CoA decarbonylase/synthase complex subunit delta	15.96	13	0.10	Acetotrophic
L0KYY9	cdhE	Acetyl-CoA decarbonylase/synthase complex subunit gamma	21.96	11	0.14	Acetotrophic
L0L0H0	mcrA	S2. reductase subunit alpha	60.77	192	4.76	CH4 formation
L0L0T9	mcrB	Methyl-coenzyme M reductase, beta subunit	60.74	98	2.99	CH4 formation
L0KWQ4	mcrG	Methyl-coenzyme M reductase, gamma subunit	66.67	55	1.60	CH4 formation
L0KYV7	mcrD	Methyl-coenzyme M reductase operon protein D	39.13	6	0.05	CH4 formation
L0KZB0	hdrA	Polyferredoxin, heterodixulfide reductase subunit A	9.80	7	0.04	CH4 formation
L0L027	hdrE	Heterodisulfide reductase subunit E	39.13	6	0.05	CH4 formation
L0L0B9	hdrD	Heterodisulfide reductase subunit D (Fe-S oxidoreductase)	23.19	11	0.11	CH4 formation
L0KWW2	cooS	Carbon-monoxide dehydrogenase, catalytic subunit	49.53	67	0.85	Miscellanceous
L0KY87		Coenzyme F420-reducing hydrogenase, beta subunit	34.40	16	0.15	Miscellanceous
L0L246		Hydrogenase maturation factor	8.37	5	0.04	Miscellanceous
L0L062		F420H2 dehydrogenase subunit FpoO	15.83	2	0.01	Miscellanceous
L0KUU0		CO dehydrogenase maturation factor	27.20	12	0.17	Miscellanceous
L0KYE3	GLUD1_2	Glutamate dehydrogenase	29.64	18	0.13	N metabolism
L0KT19	glnA	Glutamine synthetase, type I	34.16	33	0.23	N metabolism
L0KZ19	hcp	Hydroxylamine reductase	23.71	27	0.19	N metabolism
L0KU78	hcp	Hydroxylamine reductase	6.68	12	0.17	N metabolism
L0KW74		NAD(P)H-nitrite reductase	51.14	40	1.02	N metabolism

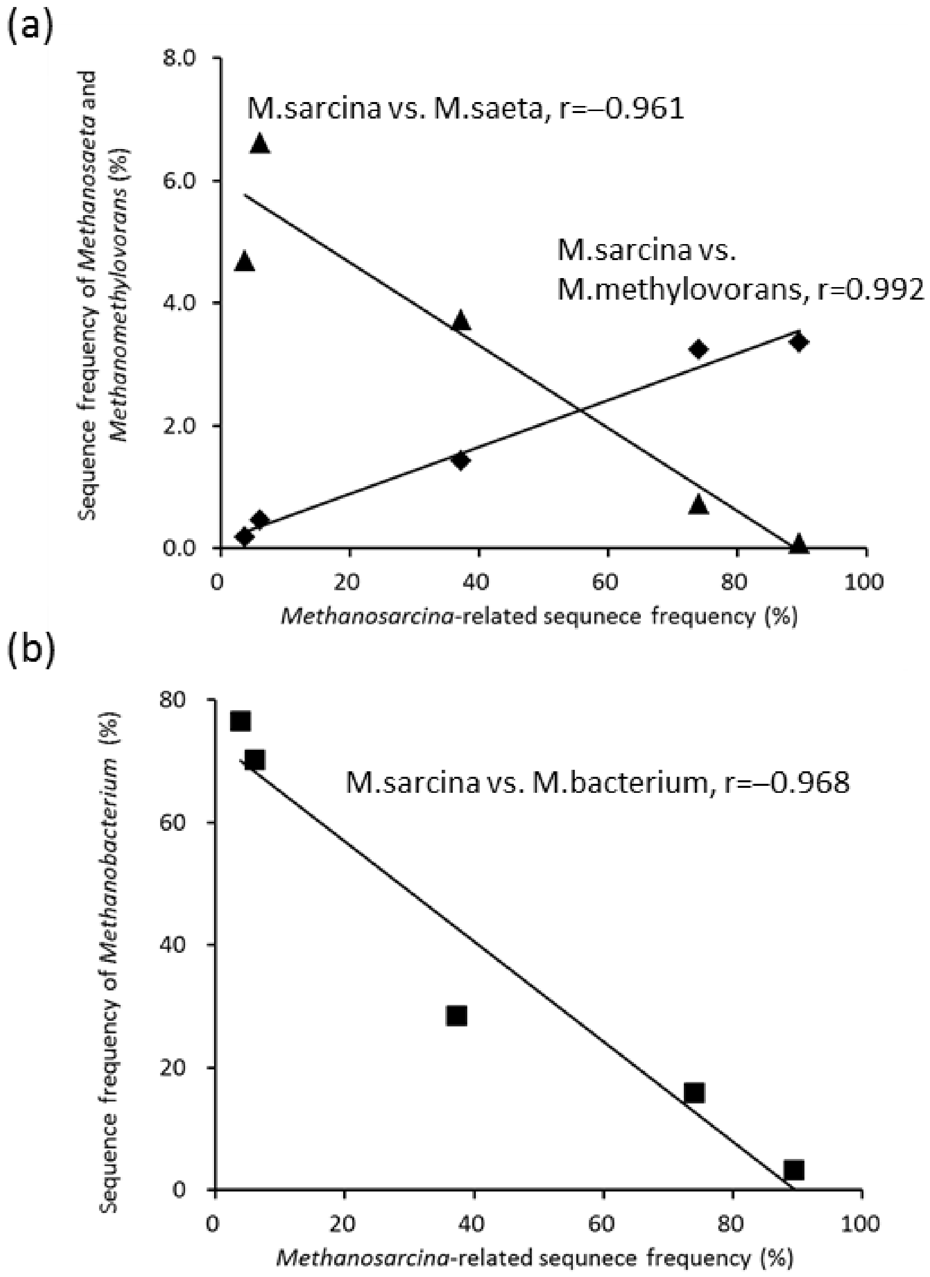

Figure S1. (a) Correlation of *Methanosarcina* vs. *Methanosaeta* and *Methanomethylovorans*. (b) Correlation of *Methanosarcina* vs. *Methanobacterium*. The lowercase, “r” denotes Pearson coefficient.

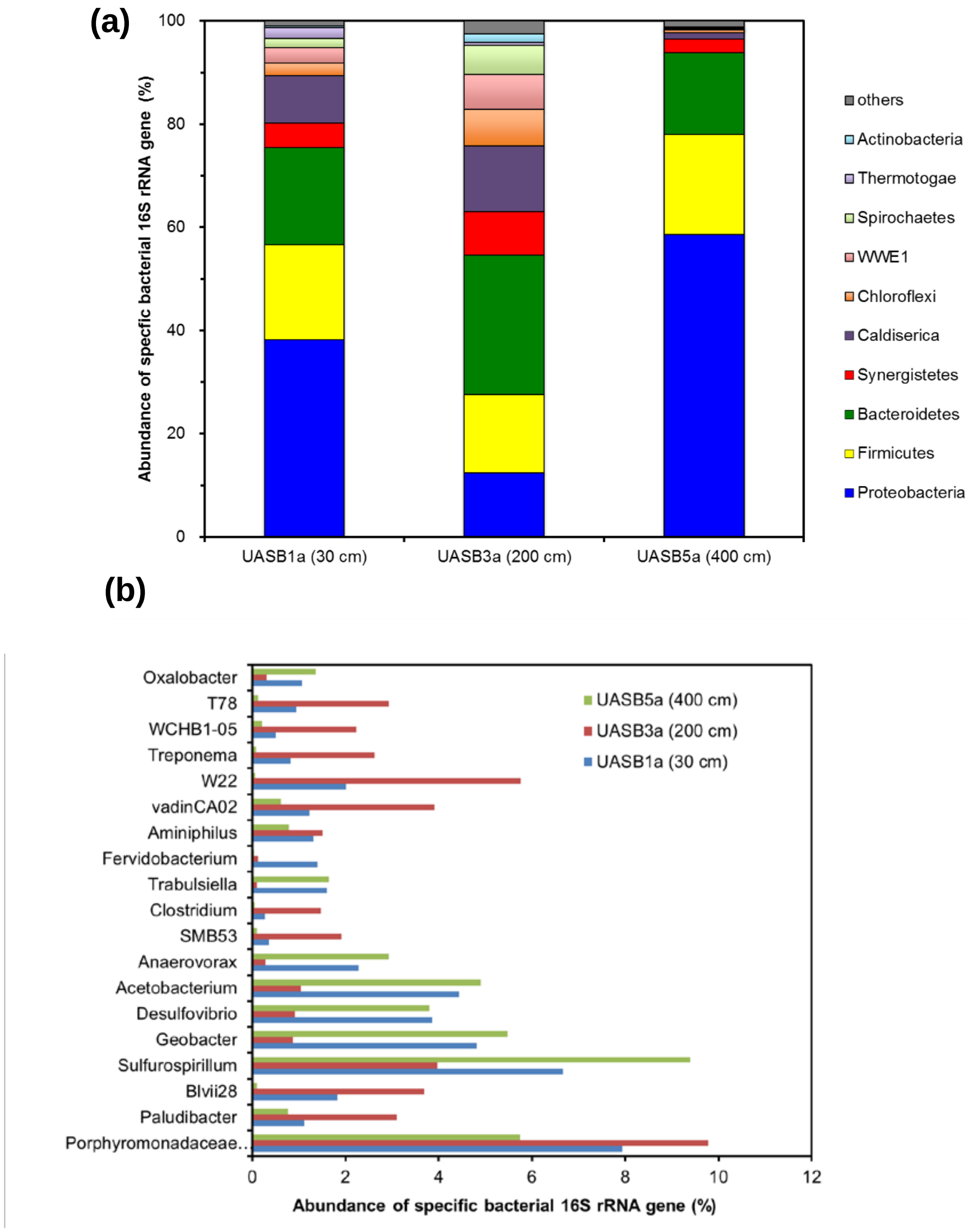

Figure S2. High-throughput sequencing analysis of bacterial 16S rRNA sequences of sludge samples taken from different heights (30cm, 200cm, and 400 cm) of a full-scale UASB reactor treating tetramethylammonium-rich wastewater. (a) Distribution of bacterial phylum-level taxa, and (b) distribution of dominant bacterial genera/groups (sequence abundance >1%).

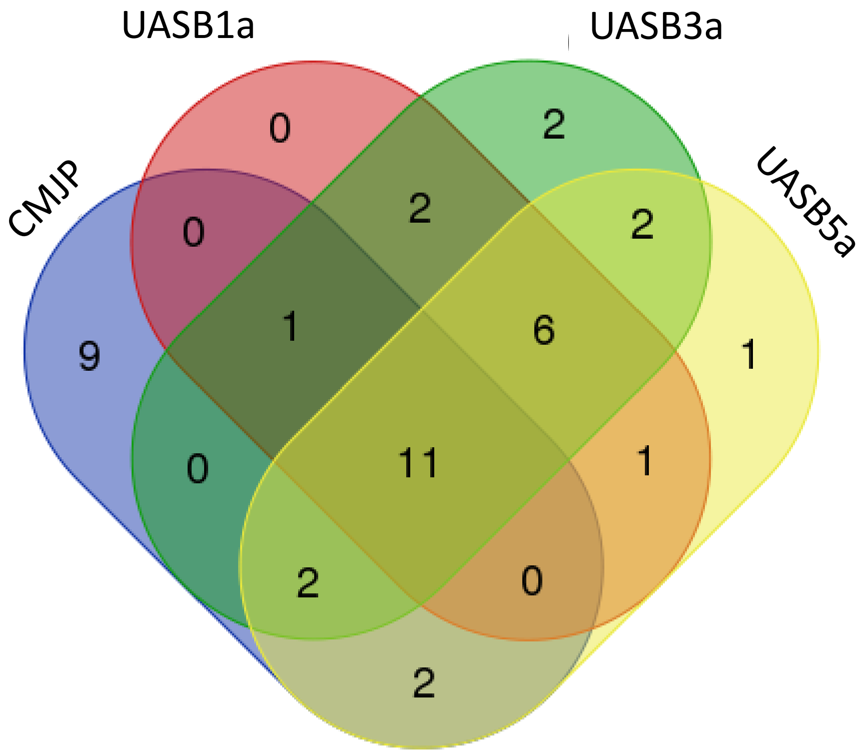

Figure S3. Venn diagram analysis of bacterial phyla detected with CMJP and UASB samples. Common phyla (shared by CMJP and UASB samples): *Proteobacteria, Spirochaetes, Synergistetes, Bacteroidetes, Acidobacteria, Thermotogae, Actinobacteria, Planctomycetes, Firmicutes, Chloroflexi,* and *Chlorobi*. CMJP specific phyla: *Hydrogenedentes, Candidatus Saccharibacteria, Ignavibacteriae, Tenericutes*, BRC1, *Elusimicrobia, Cloacimonetes, Aminicenantes*, and *Microgenomates*. UASB specific phyla: *Fusobacteria* ,WPS-2, WS6, *Lentisphaerae*, WWE1, *Caldiserica*, NKB19, OP8, OP9, OP11, TM7, OD1, TM6, and *Deferribacteres*.

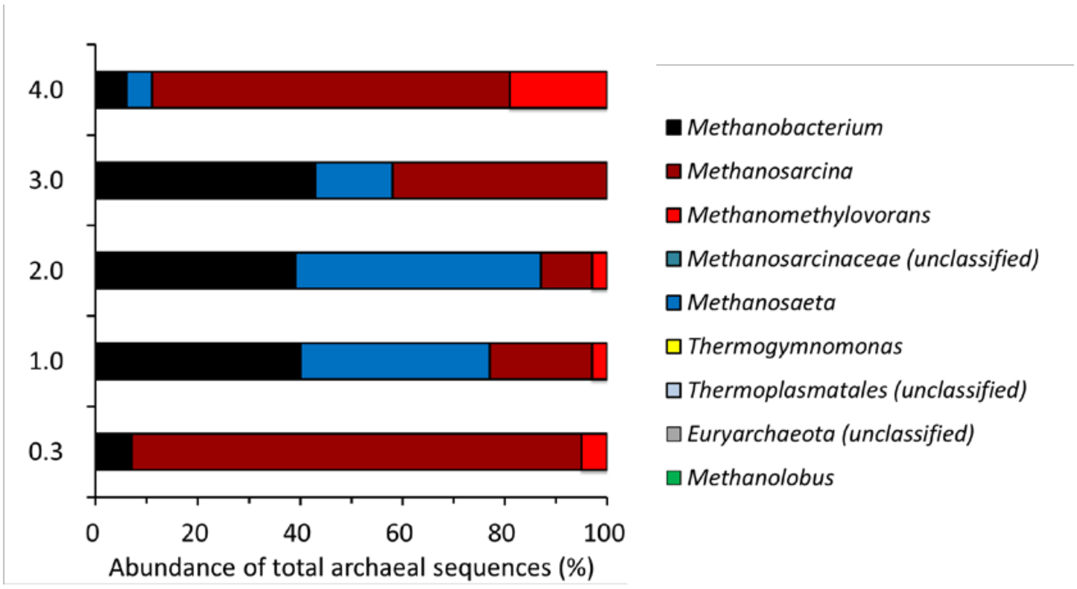

Figure S4. Quantitative distribution of archaeal populations in the full-scale UASB reactor for treating tetramethylammonium-rich wastewater as revealed by a DGGE-cloning approach.

(a)

(b)

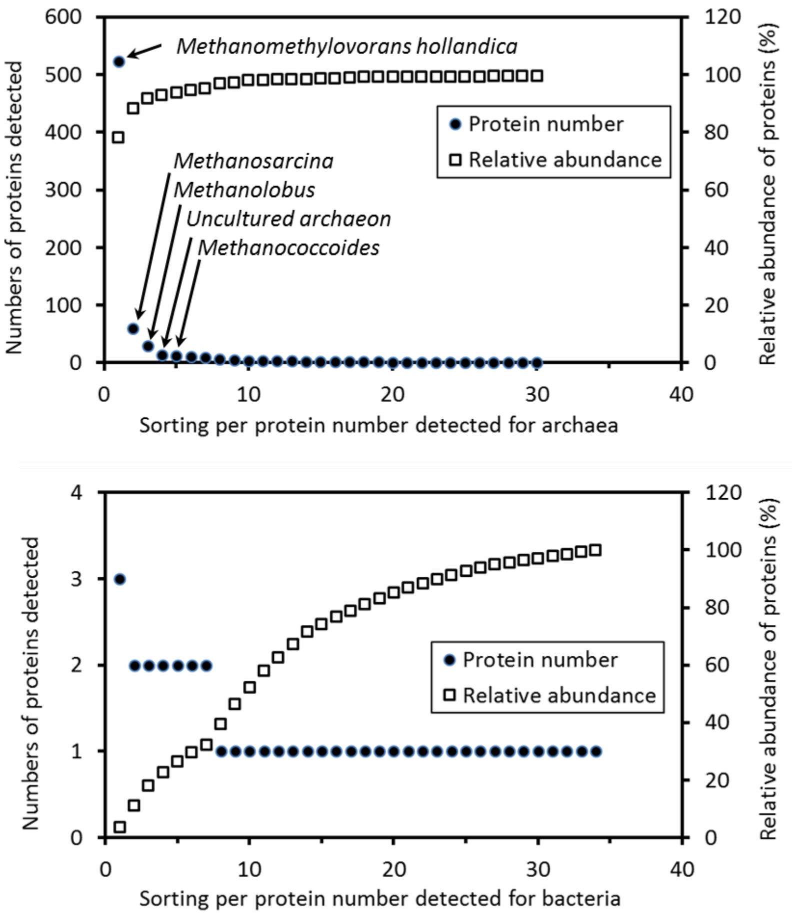

Figure S5. Numerical distribution and accumulated relative abundance of the identified proteins from (a) archaeal and (b) bacterial members in the sludge sampled from a tetramethylammonium-degrading CMSS reactor.

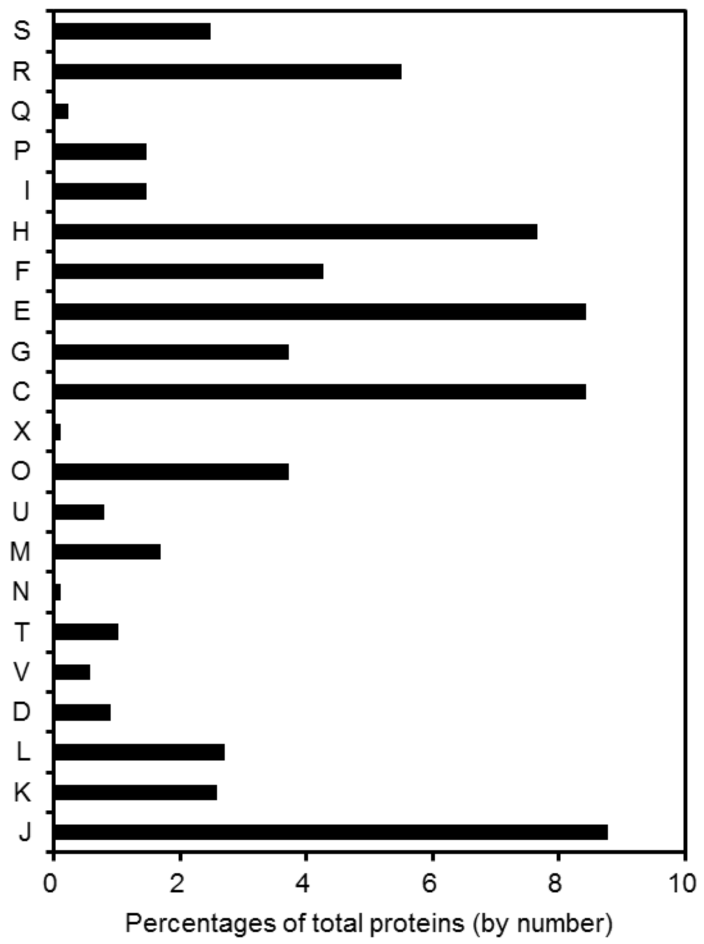

Figure S6 Distribution of the identified proteins assigned to the functional categories based on cluster of orthologous groups (COG) classification. (Symbols, **J**: Translation, ribosomal structure and biogenesis; **K**: Transcription; **L**: Replication, recombination and repair; **D**: Cell cycle control, cell division, chromosome partitioning; **V**: Defense mechanisms; **T**: Signal transduction mechanisms; **N**: Cell motility; **M**: Cell wall/membrane/envelope biogenesis; **U**: Intracellular trafficking, secretion, and vesicular transport; **O**: Posttranslational modification, protein turnover, chaperones; **X**: Mobilome: prophages, transposons; **C**: Energy production and conversion; **G**: Carbohydrate transport and metabolism; **E**: Amino acid transport and metabolism; **F**: Nucleotide transport and metabolism; **H**: Coenzyme transport and metabolism; **I**: Lipid transport and metabolism; **P**: Inorganic ion transport and metabolism; **Q**: Secondary metabolites biosynthesis, transport and catabolism; **R**: General function prediction only; **S**: Function unknown;
